# Tin Content Determination in Canned Fruits and Vegetables by Hydride Generation Inductively Coupled Plasma Optical Emission Spectrometry

**DOI:** 10.1155/2012/376381

**Published:** 2012-04-08

**Authors:** Sanda Rončević, Anica Benutić, Ivan Nemet, Buga Gabelica

**Affiliations:** ^1^Laboratory of Analytical Chemistry, Department of Chemistry, Faculty of Science, University of Zagreb, Horvatovac 102 a, 10000 Zagreb, Croatia; ^2^Croatian National Institute of Public Health, Enviromental Health Service Rockefellerova 7, 10000 Zagreb, Croatia

## Abstract

Tin content in samples of canned fruits and vegetables was determined by hydride generation inductively coupled plasma atomic emission spectrometry (HG-ICP-OES), and it was compared with results obtained by standard method of flame atomic absorption spectrometry (AAS). Selected tin emission lines intensity was measured in prepared samples after addition of tartaric acid and followed by hydride generation with sodium borohydride solution. The most favorable line at 189.991 nm showed the best detection limit (1.9 **μ**g L^−1^) and limit of quantification (6.4 **μ**g kg^−1^). Good linearity and sensitivity were established from time resolved analysis and calibration tests. Analytical accuracy of 98–102% was obtained by recovery study of spiked samples. Method of standard addition was applied for tin determination in samples from fully protected tinplate. Tin presence at low-concentration range was successfully determined. It was shown that tenth times less concentrations of Sn were present in protected cans than in nonprotected or partially protected tinplate.

## 1. Introduction

 A significant quantity of food and beverages arrive on the market in a robust form of tinplate packaging. Hermetically sealed can allow minimization of headspace oxygen and also keep a long and safe shelf life with minimal use of preservatives. However, the use of tinplate for food and beverage packing will result in some tin dissolving into food content. Tinplate corrosion depends on many factors including can material, nature of the can linings and coatings, and nature and acidity of the contacting foodstuff. Only limited reports are available on the toxicological effects of inorganic tin as present in canned foods, resultant from dissolution on the tin coating [1]. The main potential hazard from acute ingestion seems to be gastric irritation in some individuals exposed to high levels [2]. The maximum limit of 250 mg kg^−1^ for tin in canned foods and 150 mg kg^−1^ for tin in canned beverages was recommended by World Health Organization [3]. Within the European Union large discrepancies exist between national regulations and maximum permitted levels range from 50 mg kg^−1^ to 250 mg kg^−1^.

 Determination of tin in canned food became very important because it gives information about the contamination process and provides help to increase canned food quality and safety. In order to evaluate tin concentration in canned food and beverages, several analytical methods are described in the recent literature: fluorimetric with use of surfactant reagents to increase the sensitivity [4, 5], flame (FAAS) and electrothermal atomic absorption spectrometry (ETAAS) [6–8], and inductively coupled plasma optical emission spectrometry (ICP-OES) [9–11]. There are also several studies concerning the quality and resistance of tinplate after interaction with foodstuff which performed the use of X-ray microanalysis (EDS) and scanning electron microscopy (SEM) [12, 13].

 Atomic spectrometry methods in analysis of tin level in canned foods presume decomposition of food samples which is usually performed by acid digestion or dry ashing methods following by measurements in flame or graphite furnace mode of AAS or ICP-OES. Although the preparation step could influence the complete recovery of Sn, the sensitivity of applied measurement technique is also essential in correct evaluation of tin content in foods. For example, flame atomic absorption is recommended by European Committee for Standardization (CEN) as a standard analytical method for the determination of tin in fruit and vegetables preserved in cans [14]. In most of routine applications where Sn content fall below 3–5 *μ*g g^−1^, the flame AAS shows limited detection power. In combination with hydride generation system (HG), flame AAS technique becomes more sensitive, but it is greatly complicated by the dependency of the hydride species on acidity of sample solution [6, 8, 15]. Analytical performances of ICP-OES technique allow measurements of numerous elements and/or more emission lines of the particular element. Consequently, tin content could be determined in combination with other trace elements present in food samples [11, 16]. Comparable results of different sample preparation procedures followed by ICP-OES measurement of tin content were reported for matrices containing more than 30 mg kg^−1^ of tin [9]. However, interference effects in direct measurements of prepared solutions were not avoided completely, especially those caused by complex matrix. The technique of hydride generation with inductively coupled plasma optical emission spectrometry (HG-ICP-OES) is widely used to enhance the sensitivity of semimetal determination and to eliminate the majority of matrix interferences in plasma. The literature survey still suffers from the lack of data concerning the tin determination in canned food by this technique.

 In the present work a method of the tin determination in canned food samples by HG-ICP-OES method is described. The aim of study was to select the most appropriate measurement conditions for tin determination, especially at low-concentration range where FAAS shows insufficient sensitivity. Commercially available samples of fruits and vegetables analyzed by both methods were preserved in variously manufactured tinplate, that is, interior surfaces of cans were fully or partially lacquered. Among the chemical and physical factors which influence tin transfer into canned food, a quality of tinplate protection is also essential. Therefore, HG-ICP-OES as sensitive method for low-content tin determination in canned fruit and vegetable samples should be also helpful in estimating the efficiency and resistance of tinplate package.

## 2. Materials and Methods

### 2.1. Instruments

A *Prodigy High Dispersion *inductively coupled plasma optical emission spectrometer (Teledyne Leeman Labs, Hudson, NH, USA) was used for the tin content determination in all samples. The operating parameters of instrument and hydride generation device are given in Table 1. Emission lines used in this work were chosen as most prominent lines given in instrument line library file.

 A *SpectrAA 220 FS* atomic absorption spectrometer (Varian, Australia) equipped with deuterium background correction and UltrAA Sn lamp was used for flame AAS determinations. Tin analysis was performed in acetylene/nitrous oxide flame; measurements of absorption were made at 235.5 nm with 0.5 nm slit aperture.

 For the homogenization of food samples a *Mixer Büchi B400* (Büchi Labortechnik AG, Switzerland) was used; a *Hot Block CS 54* (Environmental Express, USA) was used for the digestion of previously homogenized samples.

### 2.2. Reagents and Solutions

High-purity-deionised water (Milli-Q Element system, Millipore, USA) was used for the preparation of standard solutions and dilution of samples. In the sample digestion procedures, a hydrochloric acid of suprapure grade (30% m/v) was used. Single-element standard solutions of Sn 1000 mg L^−1^ (Plasma Pure, Leeman Labs, Hudson, NH, USA) was used for the preparation of calibration standard solutions and control of plasma line positioning.

All calibration standards for AAS measurement were prepared during instrument run by automated dilution of reference tin solution of 50 mg L^−1^ in 10% v/v HCl.

 For the determination of tin by HG-ICP-OES, a fresh solution of NaBH_4_ 0.8% m/v in NaOH 0.5% m/v was prepared. Tartaric acid for the reduction of tin was prepared by dissolution of 10 g of solid compound in 1 L of ultrapure water. Calibration solutions of tin were prepared in the range of 0.1–50.0 mg L^−1^ by dilution with 1% m/v tartaric acid solution to appropriate volume. Calibration blank contained only aqueous solution of tartaric acid. Method of standard addition (MSA) included aliquots of prepared samples in which a standard solution of tin was added. MSA solutions were diluted with tartaric acid solution. The final concentration range in MSA sample solutions was 0.1–50.0 mg L^−1^ of tin.

### 2.3. Samples

 The twenty five samples of canned fruits and vegetables stored in original package at room temperature were opened, and the whole content was transferred into a *Mixer Büchi B400* where homogenization step was performed. Homogenized samples were stored in polypropylene containers until digestion step. An amount of 5 g of homogenized sample was transferred into a hot block vessel, and 10 mL of 50% v/v HCl was added. Samples were incubated in a Hot Block CS 54 at 80°C for two hours with occasional shaking of vessel content. Solution was then transferred by filtrating through filter paper (84 g/m^2^) into 50 mL volumetric flask and filled up to a volume with ultrapure water. Reagent blanks were included in each series of digestions. In order to prevent influence of oxidation and losses of analyte, samples stored in glass vessels should be submitted to measurement within 6 hours from preparation. Otherwise, they should be stored hermetically into polypropylene containers.

### 2.4. Measurement

 Atomic absorption measurements were performed in accordance with recommended standard procedure for the tin determination described in CEN/TS 15506:2007 [14]. Calibration of AAS instrument was performed with five solutions which were automatically diluted from reference standard. Control of calibration by external standard was performed after eight measuring steps, and recalibration procedure was repeated after twenty fourth measuring step. Characteristic concentration (*c*_*c*_) of tin at 1% of signal absorption was 1.24 mg L^−1^. Blank sample was measured prior to every set of samples. Each of two replicates of samples was measured three times subsequently.

**Figure 1:**
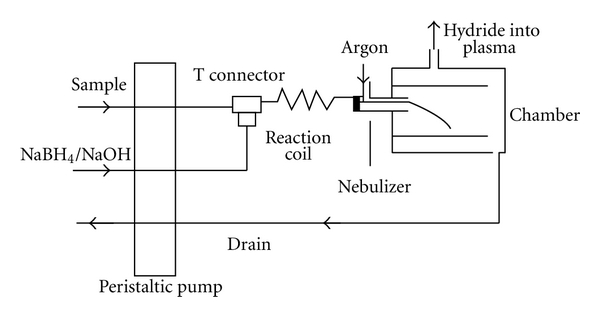
Hydride generator (HG) schematic.

**Figure 2:**
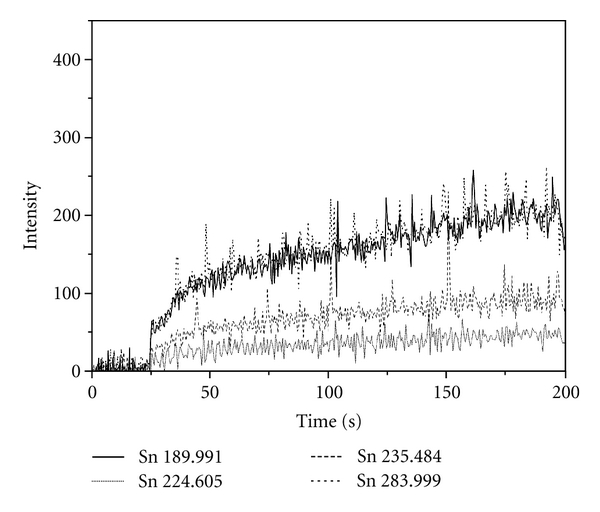
Time resolved analysis (TRA) of Sn solution (1 mg L^−1^ in tartaric acid) by HG-ICP-OES.

 For the purpose of HG-ICP-OES measurements, the calibration standards and 10-fold diluted samples were mixed with a solution of NaBH_4_ by three-channel peristaltic pump at a rate of 0.9 mL min^−1^. Hydride generator assembly scheme is shown in Figure 1. Solutions were introduced into a reaction coil where stannane gas was generated and introduced into plasma through nebulizer and nebulization chamber. The RF power and nebulizer gas flow rate were optimized to give the maximum intensity of Sn lines on L-PAD detector, that are, 1300 W and 0.9 L min^−1^, respectively. Sample uptake delay before starting of intensity measurement was set on 50 seconds. This value was selected after collecting a signal by option of time-resolved analysis (TRA) of instrument software.  Figure 2 shows signals of chosen Sn lines for solution of 1 mg L^−1^ in TRA mode. The signals started to rise after 25 seconds and plateau of signals was reached after 50 seconds from aspiration start.

**Figure 3:**
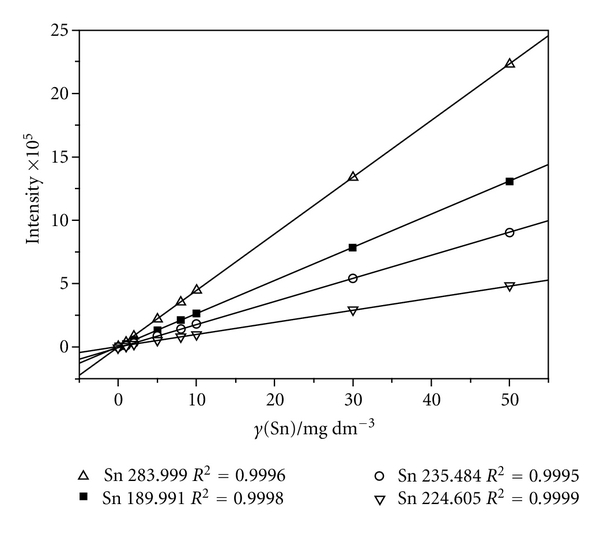
Calibration curves (HG-ICP-OES) in conventional mode.

**Figure 4:**
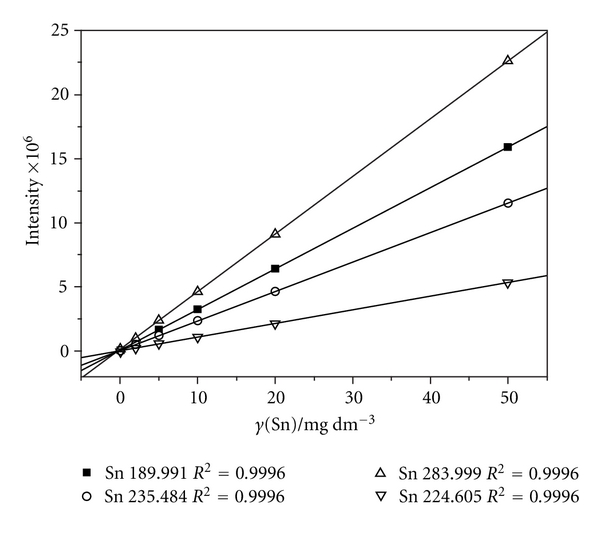
Calibration curves (HG-ICP-OES) in method of standard addition mode (MSA).

Intensity measurements were performed in triplicate. The precision of signal measurements expressed as relative standard deviation was established in the range 0.1%–4.0%. Calibration curves for each Sn line were shown in Figure 3. A good linearity of all observed Sn lines was obtained (*R* ^2^ = 0.9995–0.9999). Intensity measurement in MSA mode was performed with those samples which were initially analyzed by AAS and showed less than 5 mg L^−1^ of Sn content. An example of obtained MSA curves for a selected sample was shown in Figure 4. The intensity values in MSA operation mode were one order of magnitude greater comparing to those obtained in standard calibration. Linear coefficients comprised the same values (*R* ^2^ = 0.9996) at each observed line. The method detection limits (*c*_*L*_), which were calculated using 3*σ* criterion, that is, the concentration equivalent to three times standard deviation (3*σ*) of the signal (*n* = 10) of the reagent blank solution, are given in Table 2. The limits of quantification (LOQ) were calculated from calibration curves and expressed in *μ*g kg^−1^ of sample mass.

## 3. Results and Discussion

 HG-ICP-OES measurements of tin content in samples were performed at all selected emission lines. The precision of measurement based on RSD calculations from three replicates showed the smallest disturbance of signal at 235.484 nm (RSD 0.1%) and 189.991 nm (RSD 0.4%), compared to 283.999 nm (RSD 2.6%) and 224.605 nm (RSD 4.0%). The sensitivity of measurements should be examined from the obtained calibration curves and TRA signals. By aspirating the solution of low tin concentration, that is, 1 mg L^−1^ the better sensitivity of signal was noted at 283.999 nm and 189.991 nm lines (Figure 2). Comparison of detection capability of tin at those lines showed lower detection limit at ionic line of 189.991 nm than at atomic line of 283.999 nm. Lower detection limits, good linearity in the observed range of concentrations, and the better precision of measurements favoured the determination of tin content at 189.991 nm. Comparing with literature data, the same conclusions from ICP-OES determination of tin content without use of hydride generation were already derived [9], but an order of magnitude better sensitivity and detection capability is obtained here by use of HG-ICP-OES system. Hydride generation from samples where tartaric acid as interference-retarding agent is added also provides a selective formation of stannane gas which enters into plasma [17]. It also reduces the need to perform extensive study of possible interfering effects.

 Tin content measured by flame AAS and HG-ICP-OES in different samples of canned fruits and vegetables is shown in Table 3. All results are expressed as a milligram portion of Sn per kg of sample mass, along with standard deviation of results (*σ*). Description of tinplate protection of particular can, that is, fully or partially lacquered interior is also inserted in Table 3. Accuracy of results was tested by spiking of selected samples with Sn standard solution after homogenization step. The selected samples were foodstuffs from complete-or partially lacquered can (nos. 1 and 13). The recoveries of Sn measured by AAS were 98% for both samples. HG-ICP-OES measurement of spiked samples gave recoveries of 99% for Sample 1 and 102% for Sample 13. Statistical comparison of results obtained by reference AAS method and tested HG-ICP-OES method were performed by two-paired *t*-test at significance level of *P* = 0.05. Calculated *t*-value of 1.09 is lower than critical value of 2.06, which means that two methods were not significantly different.

 Generally, the results of flame AAS and HG-ICP-OES measurements showed that tin content in all samples did not exceed maximum permissive level of 200 mg kg^−1^ in foodstuffs. Only one sample (no. 11) of pineapples compote reached this level. Comparing the results obtained from two applied techniques, it should be noticed that for the most of samples measured by AAS and where tin content was above 5 mg kg^−1^, the similar results by HG-ICP-OES were obtained. The exceptions were samples of pineapples compote (no. 9, 10, 12, 14, and 20) and one sample of peeled plum tomatoes (No 7) with slightly higher tin concentration obtained by HG-ICP-OES. By knowing that samples for HG-ICP-OES determination include a tartaric acid which also prevents a hydrolysis of possibly present Sn (IV) species, observed difference is quite understandable. A sample of peach halves compote (no. 18) showed slightly higher Sn concentration when measured by HG-ICP-OES compared to concentration obtained by AAS. Since that longer delay from sample preparation to start of AAS measurement could lead to hydrolysis of analyte and forming of insoluble Sn (IV) compounds, a significant loss of Sn absorption signal might occur. HG-ICP-OES measurements include the use of highly reactive reducing agents which convert the majority of tin species in Sn (II) and favour the stannane gas formation. Therefore, the much more reliable value in such case is denoted to HG-ICP-OES measurement.

 Tin content in samples from complete lacquered cans (nos. 2–5, 7, 8) measured by AAS shows limitation of method capability at low-concentration range (Table 3). Considering the results where AAS measurements are limited at 5 mg kg^−1^ of tin in canned food, the method of standard addition in HG-ICP-OES technique showed exclusive advantage. Tin content measured in diluted samples was an order of magnitude higher than the LOQ of 6.4 *μ*g kg^−1^. It must be mentioned that tin determination in standard calibration mode gave approximately similar results, but with very poor precision (>20% RSD). This is mainly caused by matrix effect of solution with organic matter remains. Changes of surface tension, relative volatility, and viscosity of such solution affect the droplet distribution in HG introduction system and consequently, the amount of tin that reaches the plasma. The complexity of organic matter impact is accomplished by entering solution into plasma where changes in excitation temperature and electronic density of plasma take place. Generally, organic matter present in solution can affect the emission which results with enhancement or suppression of signal [18, 19]. Therefore, method of standard addition allows compensation of matrix effects which is also evident from the improved precision of measurements. Applied technique allows the determination of tin presence at low-concentration range where FAAS, XRF, or ICP-OES without hydride system suffer from poor sensitivity [6, 7, 9, 10, 20–22].

The results obtained for the food samples from fully protected inner walls of cans are very useful in predicting a quality of protection. It is shown that tenth times less portions of Sn are present in protected cans than in non-protected or partially protected tinplate. The exceptions, measured as approximately 70 mg kg^−1^ of tin, are established in Samples 1 and 6. The studies concerning the quality of tinplate by SEM and EDS suggest that defects of lacquer result in tin exposure [12, 13] but in much less extent than that was measured in those two samples (nos. 12, 13). Therefore, such concentrations might be attributed to the addition of tin chloride which also acts as preserving agent in manufacturing of canned foods [1, 2].

 In a summary, analysis of tin content in canned fruits and vegetables was performed by use of flame AAS which is recommended as a standard analytical method and also by HG-ICP-OES. Analytical efficiency of HG-ICP-OES method was tested on several Sn emission lines. Low-detection limit, (1.9 *μ*g L^−1^) and LOQ of 6.4 *μ*g kg^−1^, good linearity in the observed range of concentrations, and the better precision of measurements favoured the determination of tin content at 189.991 nm. Comparable results of tin content for samples from partially protected tinplate cans were obtained by use of AAS and HG-ICP-OES. Method of standard addition in HG-ICP-OES technique was applied on samples from complete lacquered cans where AAS method is limited at 5 mg kg^−1^. The reliable results for tin content at low-concentration range were established. This analytical advantage could utilize HG-ICP-OES technique as a helpful tool in certain examination of tinplate protection efficiency.

## Figures and Tables

**Table 1:** ICP-AES operating conditions.

Instrument	Prodigy high-dispersive ICP
Spectrometer	High resolution echelle polychromator
	Large-format programmable array detector (L-PAD)
RF generator	40 MHz “free running”

Argon flow	Coolant: 18 L min^−1^
	Auxiliary: 0.8 L min^−1^
	Nebulizer: 36 psi

Nebulizer	Pneumatic (glass concentric)

Spray chamber	Glass cyclonic

Hydride generator	Leeman Labs. Inc. Part No. 130-1070
	Three channel peristaltic pump (0.9 mL min^−1^)
	T connector
	Reaction coil
	Output power (1.3 kW)
	Plasma viewing (Radial)

Replicates for each analysis run	3

Sample uptake delay	50 s

Integration time	40 s

Emission lines	Sn (II) 189.991 nm
	Sn (I) 224.605 nm
	Sn (I) 235.484 nm
	Sn (I) 283.999 nm

**Table 2:** Method detection limits (*c_L_*) and limits of quantification (LOQ) for HG-ICP-OES.

Wavelength/nm	*c_L_*/*μ*g L^−1^	LOQ/*μ*g kg^−1^
Sn (II) 189.991	1.9	6.4
Sn (I) 224.605	1.4	4.5
Sn (I) 235.484	6.3	20.0
Sn (I) 283.999	6.5	20.7

**Table 3:** Tin content in canned fruit determined by AAS and HG-ICP-OES (mean of two replicate samples, *n* = 3).

No.	Sample	Tinplate protection	AAS *w*(Sn) ± *σ*/mg kg^−1^	HG-ICP-OES *w*(Sn) ± *σ*/mg kg^−1^
(1)	Peach compote, less sweat	Lacquered	68.9 ± 1.2	69.6 ± 1.1
(2)	Tomato puree, double concentrated	Lacquered	<5	2.5 ± 0.1
(3)	White beans, sterilized	Lacquered	<5	5.8 ± 0.1
(4)	Red beans, sterilized	Lacquered	<5	3.5 ± 0.1
(5)	Champignons	Lacquered	<5	0.44 ± 0.02
(6)	Champignons, sterilized	Lacquered	77.2 ± 0.8	77.2 ± 0.7
(7)	Peeled plum tomatoes, sterilized	White lacquer	<5	10.9 ± 0.2
(8)	Fruit salad	Yellow lacquer	<5	1.21 ± 0.04
(9)	Pineapples compote	Tin side and bottom, lacquered lid and seam	25.6 ± 0.1	28.8 ± 0.1
(10)	Pineapples compote	Tin side and bottom, lacquered lid and seam	37.6 ± 0.2	40.5 ± 0.2
(11)	Pineapples compote	Tin side and bottom, lacquered lid and seam	199.2 ± 3.2	199.2 ± 3.2
(12)	Pineapples compote	Tin side and bottom, lacquered lid and seam	37.7 ± 3.7	41.2 ± 3.7
(13)	Pineapples compote	Tin side and bottom, lacquered lid and seam	74.5 ± 1.1	75.5 ± 1.3
(14)	Pineapples compote	Tin side and bottom, lacquered lid and seam	23.3 ± 1.1	25.7 ± 1.1
(15)	Pineapples compote	Tin side and bottom, lacquered lid and seam	47.8 ± 0.5	47.8 ± 0.7
(16)	Pineapples compote	Tin side and bottom, lacquered lid and seam	40.3 ± 0.7	40.4 ± 0.7
(17)	Peach halves peeled, compote	Tin side and bottom, lacquered lid and seam	28.6 ± 0.5	29.6 ± 0.4
(18)	Peach halves in syrup, less sweat compote	Tin side and bottom, lacquered lid and seam	82.8 ± 1.4	104.9 ± 1.8
(19)	Compote of mixed fruits	Tin side and bottom, lacquered lid and seam	53.5 ± 0.9	54.82 ± 0.8
(20)	Pineapples compote	Tin side and bottom, lacquered lid and seam	37.7 ± 0.7	41.2 ± 0.7
(21)	Apricot compote, less sweat	Tin side and bottom, lacquered lid and seam	115.9 ± 1.4	115.6 ± 1.3
(22)	Apricot compote	Tin side and bottom, lacquered lid and seam	81.0 ± 1.1	76.7 ± 1.4
(23)	Fruit cocktail in light syrup	Tin side and bottom, lacquered lid and seam	117.3 ± 1.3	115.8 ± 1.4
(24)	Fruit cocktail in light syrup	Tin side and bottom, lacquered lid and seam	108.6 ± 1.2	107.3 ± 1.3
(25)	Mandarin oranges whole segments	Tin side and bottom, lacquered lid and seam	105.5 ± 1.3	104.8 ± 1.2
